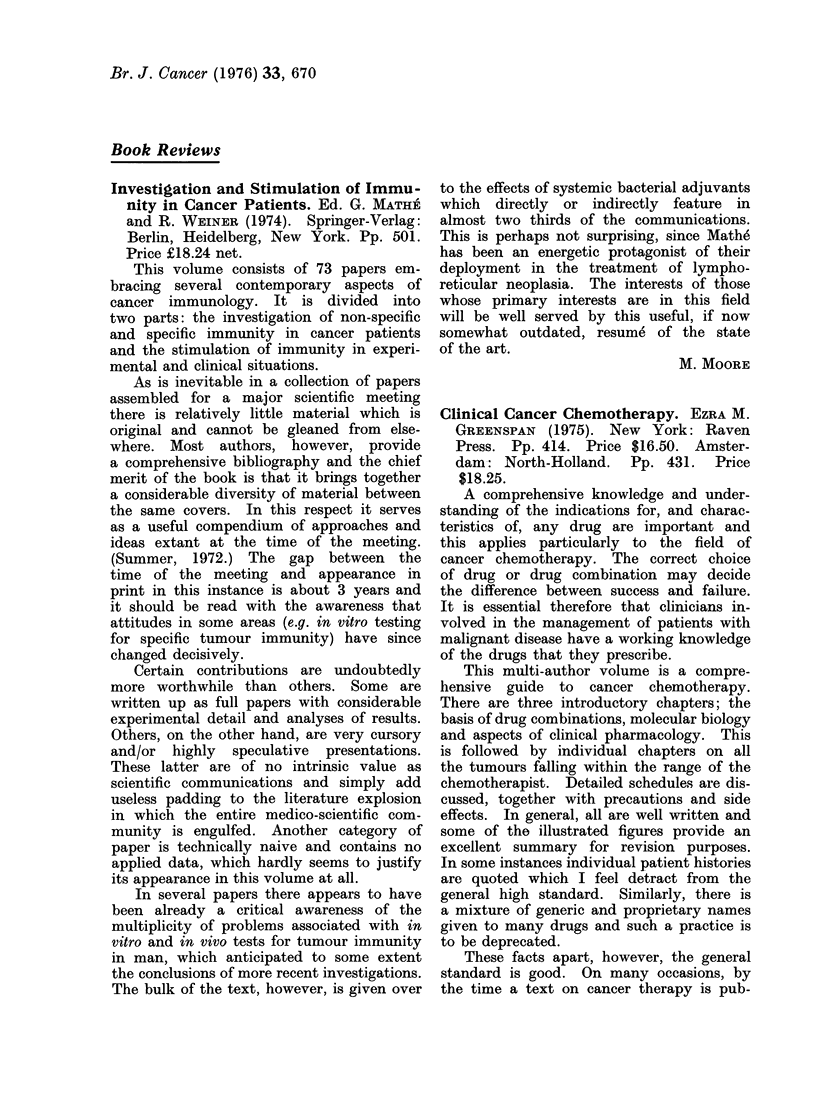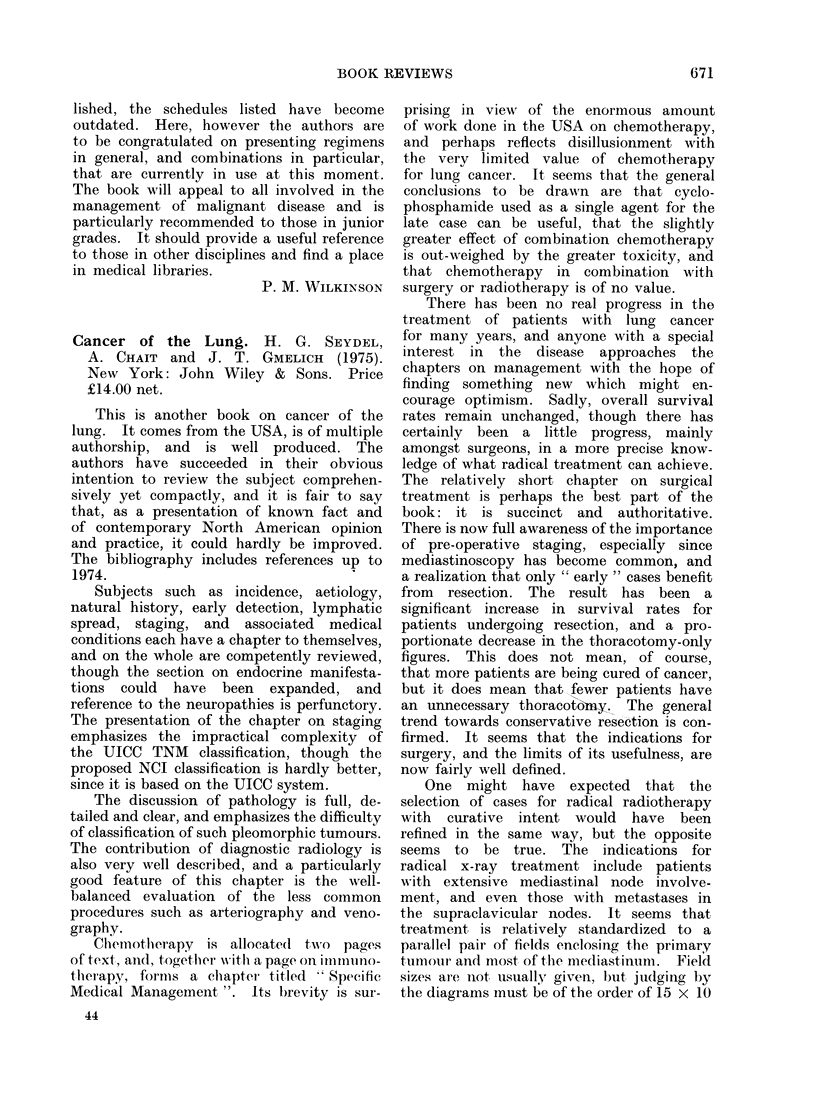# Clinical Cancer Chemotherapy

**Published:** 1976-06

**Authors:** P. M. Wilkinson


**Clinical Cancer Chemotherapy.** Ezra M. Greenspan (1975). New York: Raven Press. Pp. 414. Price $16.50. Amsterdam: North-Holland. Pp. 431. Price $18.25.

A comprehensive knowledge and understanding of the indications for, and characteristics of, any drug are important and this applies particularly to the field of cancer chemotherapy. The correct choice of drug or drug combination may decide the difference between success and failure. It is essential therefore that clinicians involved in the management of patients with malignant disease have a working knowledge of the drugs that they prescribe.

This multi-author volume is a comprehensive guide to cancer chemotherapy. There are three introductory chapters; the basis of drug combinations, molecular biology and aspects of clinical pharmacology. This is followed by individual chapters on all the tumours falling within the range of the chemotherapist. Detailed schedules are discussed, together with precautions and side effects. In general, all are well written and some of the illustrated figures provide an excellent summary for revision purposes. In some instances individual patient histories are quoted which I feel detract from the general high standard. Similarly, there is a mixture of generic and proprietary names given to many drugs and such a practice is to be deprecated.

These facts apart, however, the general standard is good. On many occasions, by the time a text on cancer therapy is published, the schedules listed have become outdated. Here, however the authors are to be congratulated on presenting regimens in general, and combinations in particular, that are currently in use at this moment. The book will appeal to all involved in the management of malignant disease and is particularly recommended to those in junior grades. It should provide a useful reference to those in other disciplines and find a place in medical libraries.

P. M. WILKINSON